# Ophthalmic manifestations and optic nerve functions in COVID-19: a prospective case series in Pakistani population

**DOI:** 10.12669/pjms.41.4.10939

**Published:** 2025-04

**Authors:** Ayisha Kausar, Sahibzada Mohammad Jan, Sulman Jaffar, Tehreem Tahir

**Affiliations:** 1 Ayisha Kausar, MCPS Ophth, FCPS Ophth Associate Professor Ophthalmology, Shifa College of Medicine, Shifa Tameer-e-Millat University, Islamabad, Pakistan; 2 Sahibzada Mohammad Jan, MBBS Demonstrator Basic Health Sciences, Shifa College of Medicine, Shifa Tameer-e-Millat University, Islamabad, Pakistan; 3 Sulman Jaffar, FCPS Ophth, FRCS Ophth Professor Ophthalmology, Shifa College of Medicine, Shifa Tameer-e-Millat University, Islamabad, Pakistan; 4 Tehreem Tahir, MBBS Post Graduate Trainee Ophthalmology, Federal Government Polyclinic Hospital, Islamabad, Pakistan

**Keywords:** Conjunctivitis, COVID-19, Optic disc edema, Visual fields

## Abstract

**Objective:**

Information on the ocular and neuro-ophthalmological manifestations of COVID-19 is relatively sparse. This study was conducted to identify optic nerve dysfunction and ocular involvement in COVID-19 patients.

**Methods:**

This prospective case series was conducted from April 2021 to December 2022 at the Ophthalmology Department of a public sector tertiary care hospital in Islamabad, Pakistan. We selected 50 patients with COVID-19 by nonprobability convenience sampling. Detailed systemic/ocular history, ocular examination and optic nerve functions were assessed. Best corrected Snellen’s acuity, color vision, contrast sensitivity, light brightness appreciation and Humphrey visual field assessments were done. Detailed slit lamp examination of anterior and posterior segment was also performed.

**Results:**

Ocular symptoms were significantly common in patients with severe systemic disease (n=50, 34% patients, *p* value0.013). The most common complaint was blurred vision (n=6, 12%). Follicular conjunctivitis was found in 60% patients. Optic nerve functions were documented for one hundred eyes of 50 patients. Seventeen eyes had color vision defects (blue yellow) and four eyes had reduced light brightness appreciation. Twenty-nine eyes had visual field defects i.e. paracentral ring scotomas, superior/inferior arcuate defects, or general reduction in sensitivity.

**Conclusions:**

One third of our study population had ocular or neuro-ophthalmic involvement. Although severity of ophthalmic involvement was related to severity of systemic COVID-19 illness and concomitant comorbid disease, ocular involvement was also reported in patients with mild systemic disease or in vaccinated patients. Prolong follow up of these patients should be considered to prevent visual complications.

## INTRODUCTION

The COVID-19 pandemic became a considerable challenge for the physicians of 21^st^ century.^1^ Declared as a global pandemic by World Health Organization in March 2020,^2^ the disease had affected 776,000,137 people and caused more than seven million deaths (till 18^th^ Aug 2024).^3^ Initially, considered an acute respiratory illness, COVID-19 later evolved as a disease affecting multiple organ systems.^4^ An ophthalmologist Dr. Li Wenliang, was among the initial physicians to monitor and subsequently succumb to COVID-19.^5^ The ocular surface/tears have been postulated as possible routes of infection transmission, although no consensus is available.^6^,^7^ The prevalence of ocular involvement in COVID-19 patients ranges from 11%-39%.^5^,^8^,^9^ Ocular and neuro-ophthalmic signs vary from mild ocular disease to severe vision-threatening complications.^8^,^10^

The estimated prevalence of neurological involvement in COVID-19 patients is 23%.^11^ Neuro-ophthalmic complications can occur in early disease or can develop during the recovery phase.^10^ Direct neurological/ early damage is caused by the virus binding to ACE-2 receptors. The pathogenesis of ocular damage and indirect neurological injury is multifactorial. It is caused by cytokine storm, proinflammatory conditions, local vascular injury, hypercoagulability and immune-mediated damage to the retina, optic nerve, and immune-mediated demyelinating injuries in the central nervous system (1-4 weeks post infection).^12^

Direct viral invasion of ocular tissue is relatively rare.^13^ The empirical/ anecdotal data on COVID-19 support ocular/ neuro-ophthalmic involvement.^5^ Majority of published articles provide only symptomatic data, collected retrospectively through survey questionnaires or from medical records, relatively little evidence is available on detailed ocular examination in COVID-19 patients. This study was conducted to describe the effect of COVID-19 on ocular health and optic nerve functions in our study population. The rationale was to inform physicians about ocular and neuro-ophthalmic complications thus, facilitating more comprehensive management strategies for COVID-19 patients.

## METHODS

This prospective case series was conducted at the Ophthalmology Department of a public sector tertiary care hospital in Islamabad, Pakistan. Fifty patients (21 males and 29 females) were enrolled through nonprobability convenience sampling from April 2021 to December 2022. Informed written consent was obtained from all participants. To minimize the risk of infection, examinations were performed after the patients were asymptomatic or after five days of isolation in asymptomatic patients.

### Ethical approval:

The study was approved by the Institutional Review Board and Ethics Committee (dated Dec 2020, IRB # 426-1246-2020).

### Inclusion criteria:

- Adult patients of both genders, 16-60 years of age, presenting within 1- 12 weeks of positive polymerase chain reaction (PCR) test for SARS-CoV-2 were included in the study. Patients with any severity of COVID and other systemic diseases without previous documented ocular involvement were also enrolled.

### Exclusion criteria:

- Patients were excluded from the study if they had previous history of amblyopia or ocular diseases that affect the ocular surface/retina/optic nerve, e.g., chronic conjunctivitis, dry eyes, keratitis, glaucoma, optic neuritis, diabetic retinopathy or optic neuropathy.

The data were collected on a predesigned proforma. Retrospective data regarding patient history, COVID-19 vaccination, duration since diagnosis, systemic illness, ocular symptoms, etc., was documented. The systemic illness was categorized as mild (self-managed at home), moderate (managed with OPD or teleconsultation) or severe (hospital admission). Prospective data included thorough slit lamp examination, and evaluation of optic nerve functions. The appearance of optic disc was evaluated on slit lamp using 78D lens. Pupil reactions were assessed subjectively by torch and slit lamp. To reduce examiner bias, all examinations were performed by single ophthalmologist.

The frequency of optic nerve function was analyzed and reported for one hundred eyes of 50 patients. The optic nerve assessment included best corrected Snellen’s visual acuity, Hardy Rand Rittler Pseudo-Isochromatic Plates (HRR) color vision, Pelli Robson contrast sensitivity, light brightness appreciation, pupil reflexes, and Humphrey visual field (VF) analysis 24-2. Dyschromatopsia was diagnosed if the patient could not read at least one of the 20 HRR plates. The contrast sensitivity was documented as the log value. Based on reliability indices unreliable VF were excluded from the results/analysis. To reduce the mask-associated field defects, analysis was performed without wearing a facemask. In our study population, 15% of the fields were unreliable.

### Statistical Analysis:

The data were analyzed by the statistical software SPSS, version 23.0. Frequencies and percentages were calculated for categorical variables, e.g., sex, ocular signs and symptoms, and type of visual field defect. For the quantitative variables, the mean ± SD was calculated, e.g., age, duration since COVID-19, mean and pattern standard deviation. The chi-square test was used to determine the association between the severity of systemic disease and ocular signs. A *p*-value < 0.05 was considered to indicate statistical significance.

## RESULTS

The mean age of our study population was 31.26±10.95 years, Range: 18-58 years. The mean interval between PCR based diagnosis of COVID-19 and ocular examination was 4.19±2.31 weeks (Range 2-10 weeks). The demographic information of the study population is shown in Table-I.

**Table-I:** Demographics of the study population and comparisons of the severity of systemic illness.

	Severity of COVID-19 systemic illness				Chi-square p-value
	Mild n=41 (82%)	Moderate n=5 (10%)	Severe n=4 (8%)	Total n=50 (%)	
Gender:					
Male	15 (36.6%)	3 (60%)	3 (75%)	21 (42%)	
Female	26 (63.4%)	2 (40%)	1 (25%)	29 (58%)	0.229
*Age groups:*					
≤20 years	2 (4.9%)	0	0	2 (4%)	
21-40 years	34 (82.9%)	4 (80%)	1 (25%)	39 (78%)	0.091
≥41 years	5 (12.2%)	1 (20%)	3 (75%)	9 (18%)	
*Ocular symptoms*					
Yes	12 (29.3%)	1 (20%)	4 (100%)	17 (34%)	0.013
No	29 (70.7%)	4 (80%)	0	33 (66%)	
*Systemic comorbid*					
*:* Yes	7 (17.1%)	0	3 (75%)	10 (20%)	0.011
No	34 (82.9%)	5 (100%)	1 (25%)	40 (80%)	
*Vaccination:*					
Vaccinated	34 (82.9%)	4 (80%)	3 (75%)	41 (82%)	0.918
Not vaccinated	7 (17.1%)	1 (20%)	1 (25%)	9 (18%)	

Majority of our patients were between 21 and 40 years of age (82.9%) and had mild disease (82%). Moderate and severe systemic signs were more common in males (*p*-value 0.229). Patients with coexisting systemic diseases (e.g., diabetes mellitus, hypertension, ischemic heart disease, asthma etc.) were significantly more prone to severe systemic illness (*p*-value 0.011). Ocular symptoms were significantly more prevalent in patients with severe systemic disease (*p*-value 0.013). The most common systemic symptoms were respiratory, i.e., cough and shortness of breath (66% of patients), followed by fever (44%) and malaise (40%) (Table-II).

**Table-II:** Frequency of systemic symptoms in the study population.

Systemic diseases	Severity of COVID-19 systemic illness			
	Mild	Moderate	Severe	Total
Respiratory	29(69.6%)	3(60%)	1(25%)	33(66%)
Fever	14(33.6%)	4(80%)	4(100%)	22(44%)
Malaise	14(33.6%)	3(60%)	3(75%)	20(40%)
Loss of taste/smell	16(38.4%)	1(20.0%)	0	17(34%)
Headache	3(7.2%)	1(20.0%)	0	4(8.0%)
Vertigo	1(2.4%)	1(20.0%)	1(25.0%)	3(6.0%)
Rhinorrhea	3(7.3%)	0	0	3(6.0%)
Gastrointestinal Symptoms	3(7.3%)	0	0	3(6.0%)
Backache	1(2.4%)	0	0	1(2.0%)

Ocular symptoms were reported by 17(34%) patients (one or both eyes) during or after their illness. The most common ocular complaint was blurred/foggy vision (12%), followed by redness (8%), eye pain (6%), diplopia (4%), burning sensations (4%), watering (4%), dryness (2%), itching (2%) and VF defects (2%). Symptoms of eye pain, redness, itching, watering and dry eyes were experienced during the first week of illness. Neuro-ophthalmic symptoms such as foggy/blurred vision, diplopia, and field defects were characteristically reported after 7-10 days of COVID-19 disease.

Conjunctival signs were the most common ocular findings, observed in at least one eye of 80% patients. These included follicles (60%), conjunctival hyperemia (26%), sub-conjunctival hemorrhages (6%), papillae (6%) and dry eyes (4%) patients. Other ocular signs were blepharitis (2%) and oval pupils/anisocoria (6%). The lens, cornea and anterior chamber were normal in all patients.

The majority of patients (84%) had a normal fundus examination. Hyperemic disc margins were present in 10% and fibrosis of small disc vessels in 6% of patients. The details of optic nerve functions are shown in Table-III. Three patients had anisocoria, and sluggish unilateral direct pupillary reflex in one eye. Light brightness appreciation was slightly reduced in four eyes (i.e., 4/5 on a self-rated scale). Color vision defects were seen in 17 eyes of 11 patients, involving blue yellow spectrum and were more common in females (*p*-value 0.045). Defective color vision was significantly more common in patients with severe COVID/hospitalization with a prior history of ischemic heart disease (*p*-value 0.004).

**Table-III:** Optic nerve examination in study population.

Clinical Parameter	Normal (N eyes)	Reduced (N eyes)	p value*
Best corrected Visual Acuity	6/6(97)	6/9(3)	0.712
H-R-R Color Vision	20/20(83)	19/20(12), 18/20(5)	0.004
Light Brightness appreciation	5/5(96)	4/5(4)	0.227
Contrast sensitivity (log value)	2.25(100)	0	--
Pupillary Reactions	Normal (97)	Sluggish direct reflex/ oval pupil (3)	0.712
Visual fields defects	No defect (56)	Visual field defect (29)	0.839

* Chi-square test *p*-value, while comparing optic nerve function with severity of systemic illness. - Frequency is reported in 100 eyes of 50 patients.

**Fig.1:**
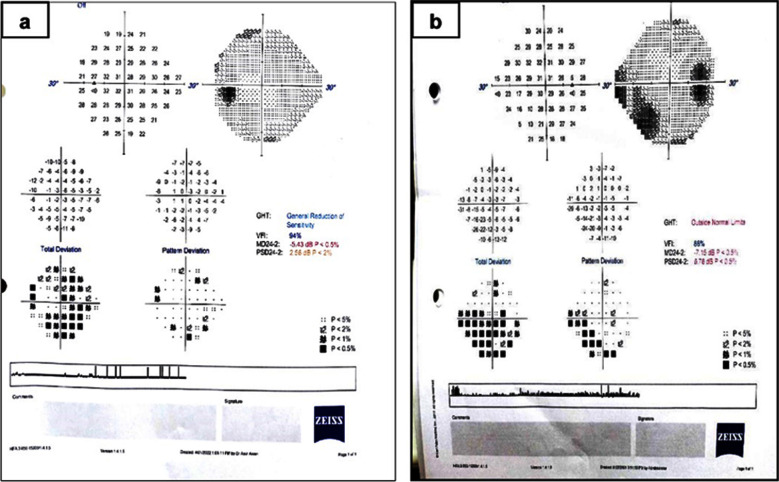
Visual field defects in COVID-19 patients. (a. 31year/ female. Fields at 10 weeks post COVID-19. Patient had bilateral general reduction of sensitivity on Humphrey field analysis, and optic disc swelling on slit lamp examination. b. 26 year /male, Fields at four weeks post COVID-19. Unilateral enlarged blind spot & inferior arcuate defect on Humphrey field analysis.)

On VF analysis 29 out of the 100 eyes had a detectable reliable VF defect. Unreliable fields (15 eyes) were excluded. The mean deviation was -1.70 ± 2.32, and the pattern standard deviation was 2.07 ± 1.65 in our study population. The mean VFI was 97.49 ± 5.02. The types of VF included paracentral ring scotoma, superior/inferior arcuate defects, blind spot enlargement and general reduction in sensitivity (Fig.1).

## DISCUSSION

This study evaluated ocular involvement in COVID-19 patients with specific emphasis on ocular and neuro-ophthalmic signs. Our study confirmed conjunctival signs in 80% of patients: follicular conjunctivitis was the most commonly observed finding. Similar findings were concluded in a meta-analysis, and conjunctivitis was observed in 88.8% patients with COVID-19.^8^ We also report a spectrum of neuro-ophthalmic signs in COVID patients. Symptoms of transient diplopia, a rare but significant finding was reported in 4% of our patients, typically presenting at 7-10 days of systemic illness with subsequent complete resolution within two weeks. No nerve palsies were observed later on examination. Tan YJ et al., also reported isolated and self-resolving ocular nerve palsies in mild cases of COVID-19.^14^ Diplopia is reported in COVID-19 patients, either in isolation or associated with systemic neurological illness.^10^,^15^ We observed unilateral oval sluggishly reacting pupil in 6% patients. Adie’s pupil diagnosis was made subjectively and not confirmed by 0.125% pilocarpine test. Cases of parasympathetic denervation/Adie’s pupil (presumed immune-mediated mechanisms) have been reported in a review study.^15^

Some studies have reported reversible visual field abnormalities in COVID-19 patients. Transient visual field abnormalities have also been reported after vaccination for viral illnesses.^16^,^17^ However, less data is available on the detailed assessment of optic nerve. Our patients had a variety of visual field defects. There was no association of field defects with vaccination (*p*-value 0.472). Sharma et al. reported reversible inferior altitudinal field defect in a 22 years old female with mild systemic illness. The visual complaints started one week after systemic COVID-19.^18^ In another case report, COVID-19 infection led to progressive deterioration of visual fields in a medically controlled pituitary macradenoma patient with subsequent improvement to baseline after two months.^19^

The contrast sensitivity was not affected in any of our patients. Dyschromatopsia (involving predominantly blue yellow spectrum) was observed in patients with severe systemic illness and a history of ischemic heart disease. A recent study concluded similar findings.^20^ This may show that optic nerve fibers from S-cone photoreceptors are affected more predominantly than L and M cones or rod photoreceptors. These findings can be linked to compromised macular blood supply in affected patients. Macular OCT scan should be considered in managing such cases.

Eye symptoms were reported by 34% (n=50) of our patients. A similar incidence was reported in a local study by Zubair et al., 38.6% of patients complained of eye problems.^9^ Their data was collected from electronic medical records of 44 patients, and mean age of study population was greater (55.75±14.53 years) than that of our patients (31.26±10.95 years). They reported watering (94.44%) as the most common symptom, followed by eye redness (33.33%) vs. redness in only 8% of our patients. Like in our study, no serious long-term visual complications were reported. In another local study ocular symptoms were reported in 64.2% patients: eye redness being the most common complaint (46%).^21^ In another international retrospective electronic survey, a higher frequency (72%) of ocular symptoms was reported (total 229 patients). Different demographics and a more intense screening process may explain higher incidence in their patients. Comparable to our study greater frequency of ocular symptoms were reported in patients with more systemic complaints (p< 0.001).^22^ In our patients, dry eyes were reported early in the course of COVID-19. In addition to virus-associated ocular surface disease, the literature shows the effect of masks on aggravating dry eye symptoms.^23^

The majority of studies on COVID-19 patients are retrospective chart reviews or self-reported online questionnaires, with inherent recall bias associated with them. This is the first study in our region in which comprehensive slit lamp examination and optic nerve evaluations were performed for all patients. Our results add substantial information to the existing body of literature on ocular involvement in COVID-19 patients.

### Limitations:

A small sample size and convenience sampling may not be true representative of the overall population and generalizations should be interpreted with caution. Patients’ enrolment during different waves of the pandemic may not be representative of one variant of COVID-19. The study lacks long-term follow-up, with limited evidence on resolution/evolution of ocular and neuro-ophthalmic findings over time.

## CONCLUSION

COVID-19 can cause a wide range of ocular and neuro-ophthalmic manifestations, affecting ocular surface, anterior and posterior segments of the eye. In addition to severe systemic disease, ocular complications can arise even in patients with mild systemic disease or in vaccinated patients. Ocular surface related complaints arise in early disease whereas neuro-ophthalmic signs emerge later during recovery phase suggesting distinct pathophysiological mechanisms. Progressive visual field defects in patients with recent COVID-19 infection should be managed meticulously.

### Author’s Contributions:

**AK, SMJ, SJ and TT:** Conception of study idea, data collection/ interpretation,

**AK and SJ:** Critical Review, Supervision of project, grant approval.

**AK, SMJ and TT:** Literature review, Manuscript drafting.

All authors have approved the final version and are accountable for the integrity of the study.
